# Clinicopathologic Significance and Immunogenomic Analysis of Programmed Death-Ligand 1 (PD-L1) and Programmed Death 1 (PD-1) Expression in Thymic Epithelial Tumors

**DOI:** 10.3389/fonc.2019.01055

**Published:** 2019-10-15

**Authors:** Joon Seon Song, Deokhoon Kim, Ji Hyun Kwon, Hyeong Ryul Kim, Chang-Min Choi, Se Jin Jang

**Affiliations:** ^1^Department of Pathology, Asan Medical Center, University of Ulsan College of Medicine, Seoul, South Korea; ^2^Center for Cancer Genome Discovery, Asan Medical Center, Asan Institute for Life Sciences, Seoul, South Korea; ^3^Samkwang Medical Laboratories, Department of Pathology, Seoul, South Korea; ^4^Department of Thoracic and Cardiovascular Surgery, Asan Medical Center, University of Ulsan College of Medicine, Seoul, South Korea; ^5^Department of Pulmonology and Medical Oncology, Asan Medical Center, University of Ulsan College of Medicine, Seoul, South Korea

**Keywords:** thymic epithelial tumor, programmed death−1, programmed death-ligand 1, immunohistochemistry, RNA-seq

## Abstract

**Objectives:** Thymic epithelial tumors (TETs) are rare malignant tumors that exhibit heterogeneous histology and clinical behavior. As immune check point inhibitors, drugs targeting anti-programmed cell death protein 1 (PD-1) and programmed death-ligand 1 (PD-L1) have shown remarkable results against many cancers; thus, the importance of PD-1/PD-L1 immunohistochemistry as a predictive or prognostic biomarker has grown. However, limited data on PD-L1 and PD-1 expression in TETs have been reported; moreover, these results have been variable. Here, we examined the expression of PD-1/PD-L1 proteins in TETs and analyzed the clinicopathologic significance of this expression.

**Patients and Methods:** A tissue microarray was constructed using 368 samples of TETs, each in triplicate. Immunohistochemistry for PD-L1 (SP263 assay) and PD-1 in TETs and CD8 in thymic carcinoma (TC) was performed; next, correlations with clinicopathologic characteristics were analyzed. PD-L1^high^ was designated as ≥50% of tumor proportion score; PD-1^high^ and CD8^high^ were defined as ≥5% and 1% of tumoral immune cells, respectively.

**Results:** The cohort consisted of 308 patients with thymomas and 60 patients with TC. PD-L1 positivity was identified in 90.6% (328/362, ≥1%) of TETs, PD-1 expression of intra-/peritumoral T cells was identified in 53.6% (194/362) of TETs and CD8 positivity was identified in 11% (7/60, ≥1%) of TC. Of the 362 patients, 141 (39.0%) exhibited high PD-L1 expression (PD-L1^high^). The PD-L1^high^ thymoma group was correlated with high Masaoka-Koga stage (*p* < 0.001), type B3 histology (*p* < 0.001), and myasthenia gravis (*p* < 0.001). This group exhibited poor overall survival (OS, *p* = 0.003, log-rank) and worse disease-free survival (DFS, *p* = 0.042, log-rank). No survival differences were detected between PD-L1^high^ and PD-L1^low^ groups in TC. Additionally, there was no correlation between PD-1 expression and survival in patients with TETs. Multivariate analysis revealed that PD-L1^high^ expression was an independent poor prognostic factor (*p* = 0.047, HR 2.087, 95% CI, 1.009–4.318) in thymomas.

**Conclusions:** To our knowledge, this is the largest study on TETs published in English literature. This study provides useful information regarding the prognosis of and potential therapeutic options for patients with TETs.

## Introduction

The thymus is primarily a lymphoid organ in which T lymphocytes mature as a component of the adaptive immune system function. As the organ ages, it undergoes involution. After maturation, several types of mature T cells, including CD4^+^ T cells (helper T cell) and CD8^+^ T cells (cytotoxic T cells, CTLs), are fully formed. CD8^+^ CTLs have immune surveillance functions that aid in eliminating cancer. Thus, the number of CD8^+^ tumor-infiltrating lymphocytes (TILs) is correlated with better survival in various cancers (1–3). Programmed death-ligand 1 (PD-L1) is an immune checkpoint protein that is expressed in tumor cells. The binding of PD-L1 to its receptor, programmed cell death protein 1 (PD-1), inhibits activated T cell proliferation in peripheral tissues leading to “T cell exhaustion,” a T cell hypo-reactive condition (4). Based on this mechanism, anti-PD-1/PD-L1 drugs have been used to treat many tumor types, including melanoma, non-small cell lung cancer (NSCLC), and head and neck cancers; the applications of these drugs have been gradually expanded to other tumor types (5–7). As these drugs are continuously used, the relevance of related biomarkers has increased. Various methods to analyze several other candidate biomarkers related to tumor immunology are under development; however, immunohistochemistry for PD-L1 is the prevalent method.

Thymic epithelial tumors (TETs), including thymoma (types A, AB, B1, B2, and B3) and thymic carcinoma (TC), are rare malignant tumors that exhibit heterogeneous histology and clinical manifestations (8, 9). Surveillance, Epidemiology, and End Results (SEER) data revealed that East Asia has a higher incidence of TETs (0.25 per 100,000 person-years) than other regions (0.08–0.15 per 100,000 person-years) (8). Known strong prognostic values include the histologic subtype, Masaoka-Koga stage, and margin status (9). Complete resection is the first choice of treatment for patients with early-stage thymoma; however, chemotherapy and radiotherapy are often used locally in advanced or metastatic disease (9). Recently, a few clinical trials on anti-PD-L1 treatment for TETs with small groups of patients have been attempted, and 22.5% of patients responded favorably (10, 11).

Nevertheless, to date, limited data on PD-L1 expression in TETs have been reported, and these results have been variable. Here, we examined PD-L1 and PD-1 expression using clinically validated antibodies and investigated TILs in TC using CD8 immunoreactivity to evaluate their clinicopathologic significance in a large TET cohort.

## Materials and Methods

### Patients and Specimen Collection

We established a cohort of 368 patients with TETs who underwent surgery and neoadjuvant and/or postoperative adjuvant radiation or chemotherapy between 1996 and 2014 at Asan Medical Center, Seoul, South Korea. All cases and the related clinical data and medical records were histologically reviewed by three pathologists (JS, JK, and SJ). Pathological diagnoses and tumor subtyping were performed according to the 2015 WHO classification of TETs (9) and the Masaoka-Koga staging system (12) using the original section. When the TETs presented with mixed histologic features, they were classified according to the predominant subtype. We excluded patients from whom only biopsy specimens were obtained and patients without clinical data.

Freshly frozen tissue samples from 26 thymomas and 16 thymic carcinomas were used for molecular validation. The bio-specimens and data used in this study were provided by the Asan Bio-Resource Center of the Korea Biobank Network [Seoul, South Korea, 2015–16(106)]. This study was approved by the ethics committee of the Asan Medical Center (approval number: 2015-965).

### Immunohistochemistry (IHC)

Tissue microarrays (TMAs) were constructed using 2-mm cores of representative tumor areas from paraffin-embedded blocks, in triplicate, to account for tumor heterogeneity. The sections were stained with an anti-PD-L1 (SP263) rabbit monoclonal primary antibody using the OptiView DAB IHC detection kit on a BenchMark XT automated staining platform, according to the manufacturer's instructions (13). Additional IHC for PD-1 (clone NAT 105, 1:1000, mouse monoclonal antibody, Cell Marque, Rocklin, CA, USA) in TETs and CD8 (clone C8/144B, 1:400, mouse monoclonal antibody, Cell Marque, Rocklin, CA, USA) in TC were performed using the same system. Normal tonsils were used as a positive control for PD-1 and CD8. Immunohistochemistry for CD8 was performed and analyzed in only TC because normal thymic tissue also contains CD8^+^ immune cells, which could register as a false positive in thymomas, especially type B.

### PD-L1, PD-1, and CD8 Scoring

Three experienced pulmonary pathologists (JS, JK, and SJ) interpreted the immunoreactivity of PD-L1, PD-1, and CD8. Tumor cells were considered positive for PD-L1 expression only if membranous or membranous and cytoplasmic staining was present (13, 14). The percentage of positive PD-L1 expression in tumor cells (tumor proportion score, TPS) was assessed, and the average of three TPSs per case was calculated. We evaluated PD-L1 expression using a scoring system with various cut-offs (1, 5, 10, 25, and 50%) based on a previously described proportion score (Cologne score) (15), as there were no established criteria for PD-L1 in TETs. The cytoplasmic and membranous staining for PD-1 was scored according to three proportion scoring categories (<5%, 5 ~10% and ≥10%), as previously described (16). IHC scoring of CD8^+^ TILS in TC was determined according to the density of cells that were positively stained for CD8; the values were classified into five proportion scoring categories (<1, ≥1, ≥5, and ≥10%), as previously described (2).

For statistical analysis, patients were divided into high and low expression groups depending on PD-L1, PD-1, and CD8 expression. The cut-off value was more than 50% for PD-L1 (denoted as PD-L1^high^), more than 5% for PD-1 (denoted as PD-1^high^), and more than 1% for CD8 (denoted as CD8^high^).

### Transcriptome Sequencing and Data Analysis

Total RNA was extracted from OCT blocks using the mirVana™ miRNA Isolation Kit (Ambion), according to the manufacturer's recommended procedures. After total RNA purification, the sample was treated with DNase using a DNA-Free kit (Ambion) to eliminate potential DNA contamination that may interfere with interpretation. RNA purity was determined by assaying 1 μl of the total RNA extract on a NanoDrop8000 spectrophotometer. Total RNA integrity was checked using a Bioanalyzer 2100 (Agilent Technologies, Palo Alto CA, USA) with an RNA Integrity Number (RIN) value. The quality and quantity of total RNA was verified using a Nanodrop1000 spectrophotometer (Thermo Scientific, Wilmington, DE, USA) and a Bioanalyzer 2100 (Agilent Technologies). The sequencing library was constructed using the TruSeq RNA Access Library preparation kit (Illumina, San Diego, USA). TruSeq RNA libraries were amplified using PCR and purified. The final product was quantified using the qualitative polymerase chain reaction (qPCR), according to the qPCR Quantification Protocol Guide, and qualified using the Bioanalyzer 2100 (Agilent Technologies). Transcriptome sequencing was conducted on the HiSeq™ 2500 platform (Illumina) at 2 × 100 bp.

Sequenced reads were aligned to the human reference genome (b37) with MapSplice, and normalized expression values were calculated with rSEM (17). Unsupervised clustering was performed with the NMF package in R (18). Gene ontology (GO) was analyzed with differentially expressed genes in DAVID (19). Immune cell populations were estimated using CIBERSORT (20).

### Statistical Analysis

Statistical analyses were performed using SPSS version 21.0 (Statistical Package for the Social Sciences, SPSS Inc., Chicago, IL, USA). Clinicopathologic variables of the PD-L1^high^ group and PD-L1^low^ group were compared using χ^2^ or Fisher's exact test for nominal variables. Overall survival and disease-free survival were compared using the Kaplan-Meier method, and the survival differences were estimated by the log-rank test. Multivariate analysis using Cox regression was performed to assess clinicopathologic variables as independent factors for survival. All *p* < 0.05 were considered statistically significant.

## Results

### Patient Characteristics

Patients with thymoma (*n* = 308) and thymic carcinoma (*n* = 60) were included in the study. The most common thymoma type was type AB (*n* = 92, 29.7%), followed by type B3 (*n* = 73, 23.5%), type B2 (*n* = 67, 21.6%), type B1 (*n* = 44, 14.2%), and type A (*n* = 32, 10.3%). The median ages were 52 years (range: 15–81 years) and 54 years (range: 28–81 years) in the thymoma and TC groups, respectively. The male to female ratio was 1.15:1 and 1.86:1 in the thymoma and TC groups, respectively. The mean tumor size was 6.3 cm (range: 0.7–16.0 cm) and 7.1 cm (range: 1.8–20.0 cm) in the thymoma and TC groups, respectively. The most common Masaoka-Koga stage of the thymomas was stage I (*n* = 195, 62.9%), followed by stage II (*n* = 69, 22.3%), stage III (*n* = 37, 11.9%), and stage IV (*n* = 7, 2.2%), whereas the most common Masaoka-Koga stage of the TCs was stage III (*n* = 20, 33.3%), followed by stage I (*n* = 15, 25%), stage IV (*n* = 13, 21.7%), and stage II (*n* = 12, 20.0%). Myasthenia gravis (MG) was present in 73 patients (19.8%) and all of these patients had a thymoma. The median follow-up period was 73 months (range: 2–237 months). Neoadjuvant treatment included chemotherapy (CTx) and radiation therapy (RTx). Neoadjuvant CTx included adriamycin, cisplatin, vincristine, and cyclophosphamide (ADOC, 2–6 cycles); ifosfamide and cisplatin (IP, 3–4 cycles); or etoposide and cisplatin (EP, 2 cycles). Neoadjuvant RTx was applied at 4,500 cGY in 25 fractions or 6,000 cGY in 30 fractions. Adjuvant therapy contained CTx and RTX. Adjuvant CTx comprised 4–6 cycles of ADOC, 4 cycles of EP, or 2–6 cycles of ifosfamide, cyclophosphamide, and etoposide (ICE). Adjuvant RTx comprised 5,040 cGY in 28 fractions.

Patient data for molecular validation are summarized in Supplemental Table 1.

### IHC Results for PD-L1, PD-1, and CD8 Expression

**Figure 1:**
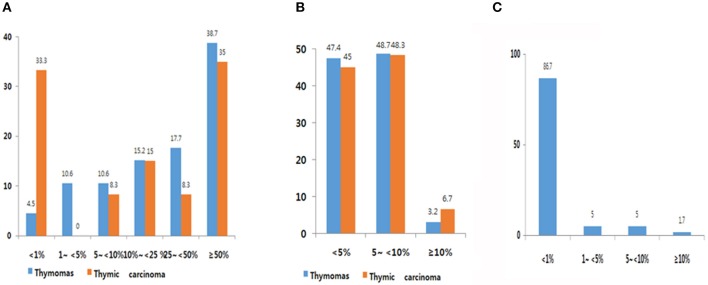
PD-L1 **(A)**, PD-1 **(B)**, and CD8 **(C)** expression frequency. **(A)** Overall, 90.6% of thymic epithelial tumors (TETs) exhibited immunopositivity for PD-L1 at cut-off of more than 1%. High PD-L1 expression was observed in 39.0% (cut-off over 50%). **(B)** Overall, 53.6% of TETs showed immunopositivity for PD-1. **(C)** Overall, 27% of thymic carcinoma samples exhibited immunopositivity for CD8.

Expression frequency for PD-L1, PD-1, and CD8 is shown in Figure 1. The average TPS of PD-L1 was 39.0 ± 31.57 in thymoma and 33.1 ± 35.95 in TC. Among 302 patients with thymomas, immuno-positivity for PD-L1 was <1%, 1–5%, 5–10%, 10–25%, and over 50% of TPS in 4.5 % (14/302), 10.6% (33/302), 10.6% (33/302), 15.2% (47/302) 17.7% (55/368) and 38.7% (120/302), respectively (Figure 1A). When compared with patients with thymomas, patients with TC (*n* = 60) had a higher proportion of those with PD-L1 <1% (33%; Figure 1B). PD-1 frequencies in thymomas and TC were similar. Of those with TC, 86.7% patients showed immunoreactivity for CD8 at <1%, whereas only one patient (1.7%, 1/60) showed immunoreactivity for CD8 at more than 10% (Figure 1C). Representative images of IHC for PD-L1 are shown in Supplemental Figure 3.

### Expression Profiling of Immune Cells

**Figure 2:**
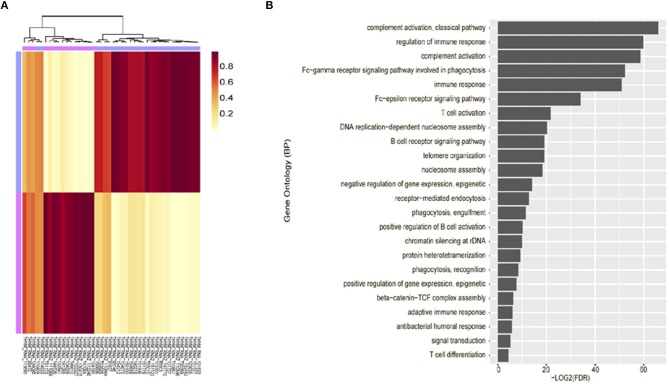
Unsupervised clustering analysis of mRNA expression profiling. **(A)** mRNA expression profiling revealed characteristic clustering patterns according to thymoma types. Cluster 1 consists of thymoma and Cluster 2 consists of thymic carcinoma. **(B)** Immune-related terms were frequently identified in gene ontology analysis.

Unsupervised clustering analysis of mRNA expression profiling was performed using 42 tumor tissues, based on the 3,000 most common genes. Two groups (cluster 1 and 2) were defined. Cluster 1 (*n* = 25) consisted of thymomas type AB to B3 and cluster 2 (*n* = 17) consisted primarily of thymic carcinoma. Thus, these were correlated with the WHO classification (*p* = 1.021 × 10^−10^, Fisher exact test, Figure 2A).

**Figure 3:**
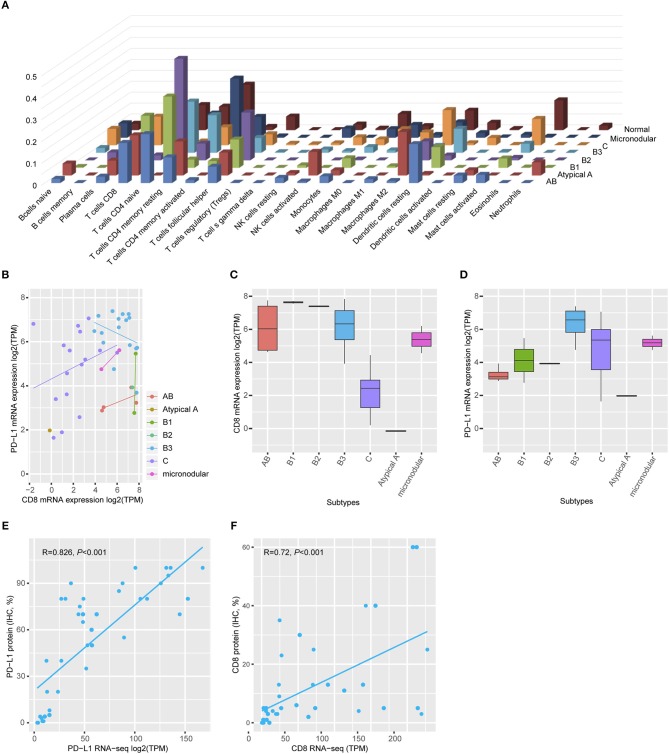
Immune cell profiling. **(A)** Plasma cells and macrophages were frequently identified in the thymic carcinoma, and CD4^+^ and CD8^+^ T-cells were present in the thymomas. Normal thymus tissue predominantly expressed CD4^+^ cells. **(B)** mRNA levels of CD8 and PD-L1 differed according to tumor subtype. **(C)** Type AB exhibited high CD8 mRNA levels. **(D)** Types B3 and thymic carcinoma exhibited higher PD-L1 mRNA expression than that of a normal thymus. Maximum PD-L1 expression in a normal thymus = 4.69 transcripts per million (TPM). The cut-off value is log2 (TPM) ≥5. **(E,F)** Both PD-L1 (e, Spearman correlation *r* = 0.826, *p* < 0.001) and CD8 (f, Spearman correlation *r* = 0.726, *p* < 0.001) protein expression correlated with mRNA level.

Gene ontology analysis revealed that differentially expressed genes between two groups were enriched for immune-related terms (Figure 2B). During immune cell population profiling according to tumor type, plasma cells and macrophages were more frequently observed in thymic carcinomas, whereas CD4^+^ and CD8^+^ T cells and dendritic cells were more frequently observed in the thymomas compared to the normal thymus (Figure 3A). mRNA levels of CD8 and PD-L1 differed according to tumor subtype (Figure 3B). PD-L1 and CD8 mRNA expression profiling analysis revealed that micronodular type, type B3, and thymic carcinoma were correlated with higher PD-L1 mRNA expression (Figure 3D); moreover, type A (atypical) and thymic carcinoma were correlated with lower CD8 mRNA expression (Figure 3C). Thus, patients with type B3, micronodular type, and thymic carcinoma would likely benefit from anti-PD-L1 immunotherapy based on PD-L1 mRNA expression profiling.

### Correlation Between Protein Expression by IHC and mRNA Expression of PD-L1

To evaluate the correlation between protein expression and mRNA levels, an additional TMA was constructed with paired cases for transcriptome analysis using formalin-fixed paraffin-embedded tumor blocks. IHC analysis revealed that PD-L1 protein expression was positively correlated with PD-L1 mRNA expression (Spearman correlation analysis, *r* = 0.826, *p* < 0.001, Figure 3E). Moreover, CD8 protein expression was also positively correlated with CD8 mRNA expression (Spearman correlation analysis, *r* = 0.726, *p* < 0.001, Figure 3F). Thus, PD-L1 and CD8 IHC analysis accurately reflected the mRNA levels.

### Correlation Between PD-L1 and PD-1 Expression With Clinicopathologic Characteristics

Of 308 patients with thymomas, 121 (38.7%) were classified as PD-L1^high^ and 181 (61.3%) patients were classified as PD-L1^low^. The PD-L1^high^ group was correlated with male predominance (*p* = 0.003), higher Masaoka-Koga staging (*p* < 0.001), type B3 (*p* < 0.001), absence of capsule formation (*p* = 0.046), presence of capsule invasion (*p* < 0.001), presence of MG (*p* < 0.001), neoadjuvant chemotherapy group (*p* = 0.033), and adjuvant radiation therapy group (*p* = 0.002). However, other parameters, including tumor size (*p* = 0.138), lymph node metastasis (*p* = 0.083), and distant metastasis (*p* = 0.164), were not correlated with PD-L1^high^ classification. These results are summarized in Table 1.

**Table 1:** Correlation between PD-L1 and PD-1 expression and clinicopathologic parameters in thymomas.

**Parameters**	**PD-L1 (*****N*** **=** **302)**			**PD-1 (*****N*** **=** **308)**		
	**High (*n =* 121, %)**	**Low (*n =* 181, %)**	***P*-value**	**High (*n =* 161, %)**	**Low (*n =* 147, %)**	***P*-value**
**Age**^**†**^	51.72 ± 13.16	51.61 ± 13.17	0.905	52.12 ± 13.23	51.05 ± 13.37	0.479
**Gender**			0.003			0.295
Male	73 (60.3%)	81 (44.8%)		78 (48.4%)	80 (54.4%)	
Female	48 (39.7%)	100 (55.2%)		83 (51.6%)	67 (45.6%)	
**Masaoka stage**			<0.001			0.012
I	59 (48.8%)	130 (71.8%)		116 (72.0%)	79 (53.7%)	
IIa	22 (18.2%)	29 (16.0%)		22 (13.7%)	29 (19.7%)	
IIb	11 (9.1%)	7 (3.9%)		8 (5.0%)	10 (6.8%)	
III	23 (19.0%)	14 (7.7%)		11 (6.8%)	26 (17.7%)	
IVa	1 (0.8%)	0 (0.0%)		1 (0.6%)	0 (0%)	
IVb	5 (4.1%)	1 (0.6%)		3 (1.9%)	3 (2.0%)	
**WHO classification**			< 0.001			0.061
A	9 (7.4%)	23 (10.4%)		13 (8.1%)	19 (12.9%)	
AB	10 (8.3%)	80 (44.2%)		62 (38.5%)	30 (20.4%)	
B1	8 (6.6%)	35 (19.3%)		29 (18.0%)	15 (10.2%)	
B2	37 (30.6%)	28 (15.5%)		26 (16.1%)	41 (27.9%)	
B3	57 (47.1%)	15 (8.3%)		31 (19.3%)	42 (28.6%)	
**Size**			0.138			0.068
<5 cm	48 (39.7%)	56 (30.9%)		63 (39.1%)	43 (29.3%)	
≥5 cm	79 (60.3%)	125 (69.1%)		98 (60.9%)	104 (70.7%)	
**Capsule formation**			0.046			0.568
Present	50 (41.3%)	96 (53.0%)		73 (49.7%)	74 (46.0%)	
Absent	71 (58.7%)	85 (47.0%)		74 (50.3%)	87 (54.0%)	
**Capsule invasion**			< 0.001			0.5106
Present	49 (40.5%)	35 (19.3%)		46 (28.6%)	38 (25.9%)	
Absent	72 (59.5%)	145 (80.7%)		115 (71.4%)	108 (73.5%)	
**LN metastasis**			0.083			0.576
Present	2 (1.7%)	0 (0.0%)		1 (6%)	1 (0.7%)	
Absent	119 (98.3%)	181 (100%)		160 (99.4%)	145(98.6%)	
**Distant metastasis**			0.164			0.380
Present	6 (5.0%)	3 (1.7%)		6 (3.7%)	3 (2.0%)	
Absent	115 (95.0%)	178 (98.3%)		155 (96.3%)	144 (98.0%)	
**Myasthenia gravis**			<0.001			<0.001
Present	51 (42.1%)	22 (12.2%)		25 (15.5%)	48 (32.7%)	
Absent	70 (57.9%)	159 (87.8%)		136 (84.5%)	99 (67.3%)	
**Neoadjuvant Tx**			0.033			0.788
No	108 (89.3%)	175 (96.7%)		152 (94.4%)	137 (93.2%)	
CTx	11 (9.1%)	5 (2.8%)		8 (5.0%)	8 (5.4%)	
RTx	2 (1.7%)	1 (0.6%)		1 (1.4%)	2 (1.4%)	
**Adjuvant Tx**			0.002			0.461
No	67 (55.4%)	135 (74.6%)		111 (68.9%)	96 (65.3%)	
CTx	2 (1.7%)	2 (1.1%)		3 (1.9%)	1 (0.7%)	
RTx	52 (43.0%)	44 (24.3%)		47 (29.2%)	50 (34.0%)	
**PD-1**			0.197			
High	58 (36.5%)	63 (44.1%)				
Low	101 (63.5%)	80 (55.9%)				

† Analyzed using a t-test.

*Tx, treatment; CTx, chemotherapy; RTx, radiation therapy*.

Of 308 patients with thymomas, 161 (51.9%) patients were classified as PD-1^high^ and 147 (47.4%) were classified as PD-1^low^. PD-1^high^ was correlated with a lower Masaoka-Koga stage (*p* = 0.012) and an absence of MG (*p* < 0.001). The correlation between PD-L1 and PD-1 expression was not statistically significant (*p* = 0.197) in thymomas. These results are summarized in Table 1.

Of 60 patients with TC, both PDL-1 and PD-1 were not correlated with any of the clinicopathologic parameters (Table 2). In addition, CD8 expression also showed the same results as PD-L1 and PD-1 (Table 3).

**Table 2:** Correlation between PD-L1 and PD-1 expression and clinicopathologic parameters in thymic carcinoma.

**Parameters**	**PD-L1 (*****N*** **=** **60)**			**PD-1 (*****N*** **=** **60)**		
	**High (*n =* 21, %)**	**Low (*n =* 39, %)**	***P*-value**	**High (*n =* 33, %)**	**Low (*n =* 27, %)**	***P*-value**
**Age**^**†**^	56.0 ± 9.41	55.69 ± 12.10	0.433	58.0 ± 11.80	53.11 ± 985	0.091
**Gender**			0.573			0.183
Male	15 (71.4%)	24 (61.5%)		19 (57.6%)	20 (74.1%)	
Female	6 (28.6%)	15 (38.5%)		14 (42.4%)	7 (25.9%)	
**Masaoka stage**			0.835			0.644
I	5 (23.8%)	10 (25.6%)		10 (30.3%)	5 (18.5%)	
IIa	2 (9.5)	7 (17.9%)		6 (18.2%)	3 (11.1%)	
IIb	1 (4.8%)	2 (5.1%)		1 (3.0%)	2 (7.4%)	
III	7 (33.3%)	13 (33.3%)		9 (27.3%)	11 (40.7%)	
IVa	2 (9.5%)	1 (2.6%)		1 (3.0%)	2 (7.4%)	
IVb	4 (19.0%)	6 (15.4%)		6 (18.2%)	4 (14.8%)	
**Size**			0.218			0.554
<5 cm	3 (14.3%)	12 (30.8%)		7 (21.2%)	8 (29.6%)	
≥5 cm	18 (85.7%)	27 (69.2%)		26 (78.8%)	19 (70.4%)	
**Capsule formation**			0.404			0.903
Present	1 (4.8%)	6 (15.4%)		4 (14.1%)	3 (11.2%)	
Absent	10 (95.2%)	33 (84.6%)		29 (87.9%)	24 (88.9%)	
**Capsule invasion**			0.866			0.599
Present	13 (61.9%)	25 (64.1%)		22 (66.7%)	16 (59.3%)	
Absent	8 (38.1%)	14 (35.9%)		11 (33.3%)	11 (40.7%)	
**LN metastasis**			0.606			0.620
Present	2 (9.5%)	2 (5.1%)		3 (9.1%)	1 (3.7%)	
Absent	19 (90.5%)	37 (94.9%)		30 (90.9%)	26 (94.9%)	
**Distant metastasis**			0.622			0.521
Present	5 (23.8%)	7 (18.4%)		8 (24.2%)	4 (15.4%)	
Absent	16 (76.2%)	31 (81.6%)		25 (75.8%)	22 (84.6%)	
**Myasthenia gravis**			None			None
Present	0 (0%)	0 (0%)		0 (0%)	0 (0%)	
Absent	21 (100%)	39 (100%)		33 (100%)	27 (100%)	
**Neoadjuvant Tx**			0.149			0.262
No	11 (6.7%)	30 (76.9%)		24 (72.7%)	17 (63.0%)	
CTx	9 (42.9%)	8 (20.5%)		9 (27.3%)	8 (29.6%)	
RTx	1 (4.8%)	1 (2.6%)		0 (0%)	2 (7.4%)	
**Adjuvant Tx**			0.934			0.578
No	6 (28.6%)	11 (28.2%)		10 (30.3%)	7 (25.9%)	
CTx	3 (14.3%)	7 (17.9%)		4 (12.1%)	6 (22.2%)	
RTx	12 (57.1%)	21 (53.8%)		19 (57.6%)	14 (51.9%)	
**PD-1**			0.277			
High	14 (42.4%)	7 (25.9%)				
Low	19 (57.6%)	20 (74.1%)				

† Analyzed using a t-test.

*Tx, treatment; CTx, chemotherapy; RTx, radiation therapy*.

**Table 3:** Correlation between CD8 expression and clinicopathologic parameters in thymic carcinoma.

**Parameters**	**CD8 (*****N*** **=** **59)**		
	**High** **(*n =* 4, %)**	**Low** **(*n =* 55, %)**	***P*-value**
**Age^*†*^**	52.8 ± 18.30	56.1 ± 10.79	0.037
**Gender**			0.647
Male	3 (75.0%)	35 (63.6%)	
Female	1 (25.0%)	20 (36.4%)	
**Masaoka stage**			0.780
I	2 (50.0%)	13 (23.6%)	
IIa	1 (25.0)	8 (14.5%)	
IIb	0 (0%)	3 (5.5%)	
III	1 (25.0%)	19 (34.5%)	
IVa	0 (0%)	2 (3.6%)	
IVb	0 (0%)	10 (18.2%)	
**Size**			0.248
<5 cm	0 (0%)	14 (25.5%)	
≥5 cm	4 (100%)	41 (74.5%)	
**Capsule formation**			0.447
Present	0 (0%)	7 (12.7%)	
Absent	4 (100%)	48 (87.3%)	
**Capsule invasion**			0.124
Present	1 (25.0%)	37 (67.3%)	
Absent	3 (75.0%)	18 (32.7%)	
**LN metastasis**			0.576
Present	0 (0%)	4 (7.3%)	
Absent	4 (100%)	51 (92.7%)	
**Distant metastasis**			0.290
Present	0 (0%)	12 (22.2%)	
Absent	4 (100%)	42 (77.8%)	
**Myasthenia gravis**			None
Present	0 (0%)	0 (0%)	
absent	4 (100%)	55 (100%)	
**Neoadjuvant Tx**			0.546
No	2 (50.0%)	39 (70.9%)	
CTx	2 (50.0%)	14 (25.5%)	
RTx	0 (0%)	2 (3.6%)	
**Adjuvant Tx**			0.580
No	1 (25.0%)	16 (29.1%)	
CTx	0 (0%)	10 (18.2%)	
RTx	3 (75.0%)	29 (52.7%)	
**PD-L1**			0.481
High	2 (50.0%)	18 (32.7%)	
Low	2 (50.0%)	37 (67.3%)	

† Analyzed using a t-test.

*Tx, treatment; CTx, chemotherapy; RTx, radiation therapy*.

### Survival Outcomes

**Figure 4:**
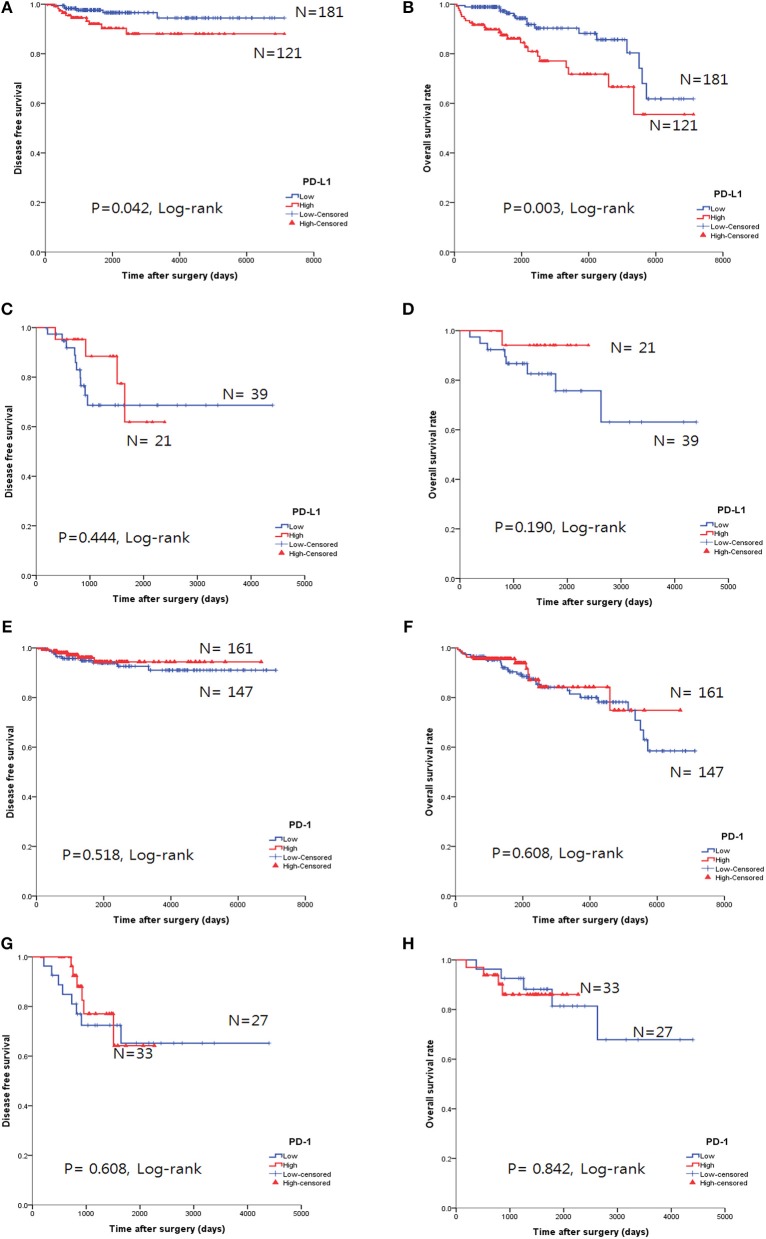
Survival analysis using the Kaplan-Meier method according to PD-L1 and PD-1 expression. **(A,B)** The PD-L1^high^ group correlated with poor overall survival (OS, **B**) and disease-free survival (DFS, **A**) in thymomas. **(C,D)** No survival differences, according to PD-L1 expression, were observed in thymic carcinoma. **(E–H)** No survival differences in the status of PD-l expression were identified in either thymomas **(E,F)** or thymic carcinoma **(G,H)**.

The overall survival rate (OS) and disease-free survival rate (DFS) are shown in Figure 4. In thymomas, the PD-L1^high^ group exhibited poorer DFS (*p* = 0.042, log-rank test, Figure 4A) and OS (*p* = 0.003, log-rank test, Figure 4B) than the PD-L1^low^ group; no differences in DFS (*p* = 0.444, log-rank test, Figure 4C) and OS (*p* = 0.190, log-rank test, Figure 4D) were observed between the two groups in TC. Interestingly, the PD-L1^high^ group in TC tended to exhibit good OS, although this trend was not statistically significant.

PD-1 expression was not related to survival differences in thymomas and TC (Figures 4E–H). Analysis of the PD-L1 and PD-1 expression combinations in thymomas revealed that PD-L1^low^/PD-1^high^ groups exhibited good OS (*p* = 0.009, log-rank, Supplemental Figure 1B) compared to PD-L1^high^/PD-1^low^ group; however, no differences in DFS were observed (*p* = 0.082, log-rank, Supplemental Figure 1A).

**Figure 5:**
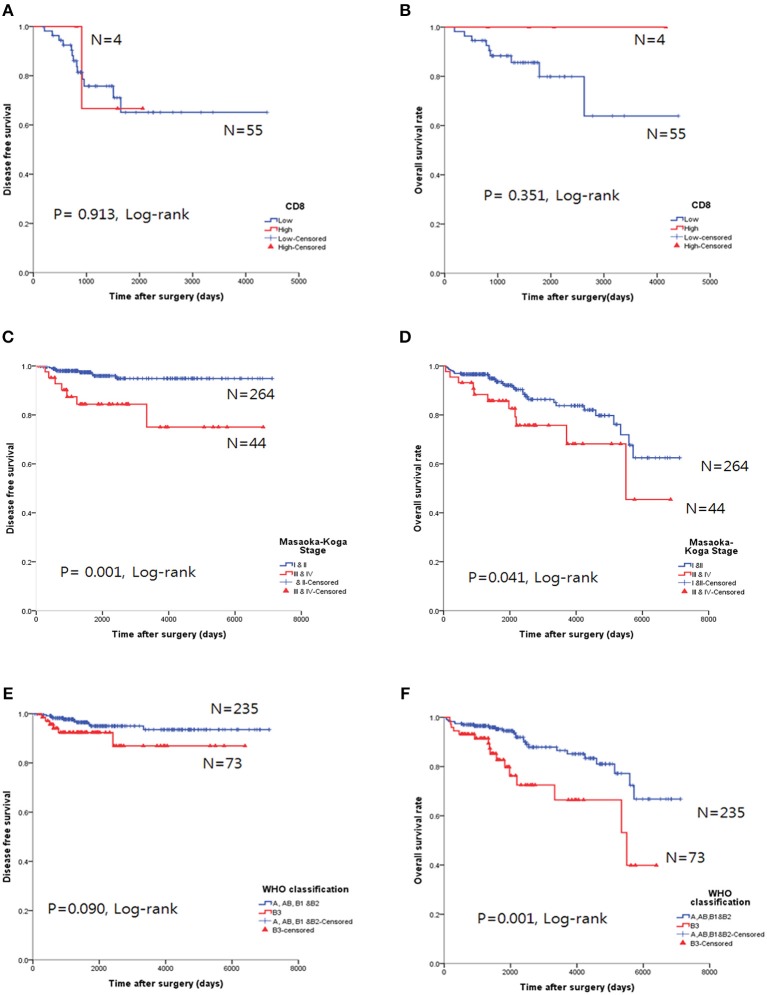
Survival analysis using the Kaplan-Meier method. **(A,B)** The CD8^high^ group was not correlated with either overall survival (OS, **A**) or disease-free survival (DFS, **B**) in thymic carcinoma. **(C,D)** Higher Masaoka-Koga stage in thymoma was correlated with both poor OS and DFS. **(E,F)** Type B3 was correlated with poor OS in thymomas; however, DFS was not significant.

The CD8^high^ group in TC tended to exhibit good OS, but the results were not statistically significant for OS (*p* = 0.351, log-rank, Figure 5A) and DFS (*p* = 0.913, log-rank, Figure 5B).

Higher Masaoka-Koga stages (*p* = 0.041, log-rank test, Figure 5D) and type B3 group (*p* = 0.001, log-rank, Figure 5F) in thymomas presented poor OS prognosis. In the univariate analysis, Masaoka-Koga stage (*p* = 0.041), WHO classification (*p* = 0.001), PD-L1^high^ (*p* = 0.003), neoadjuvant treatment (*p* = 0.002), and adjuvant treatment (*p* < 0.001) were statistically significant (data not shown). However, in the multivariate analysis, PD-L1^high^ was an independent poor prognostic factor for OS (*p* = 0.047, HR 2.09, 95% CI, 1.009–4.318) of thymomas. These findings are shown in Table 4.

**Table 4:** Multivariate analysis for overall survival in thymomas.

**Parameter**		***P*-value**	**HR**	**95% CI**
Masaoka-Koga stage	[I, II] vs. III, IV	0.617	1.266	0.503–3.186
WHO classification	[A, AB, B1, B2] vs. B3	0.065	2.063	0.956–4.449
Neoadjuvant Tx	[none] vs. CTx	0.507	0.599	0.132–2.719
	[None] vs. RTx	0.151	3.382	0.642–17.832
Adjuvant Tx	[None] vs. CTx	0.003	9.763	2.189–43.552
	[None] vs. RTx	0.086	0.477	0.205–1.110
PD-L1	[Low] vs. high	0.047	2.087	1.009–4.318

[] represents the reference parameters. HR represents hazard ratio. CI represents confidence interval.

*Tx, treatment; CTx, chemotherapy; RTx, radiation therapy*.

## Discussion

We evaluated PD-L1, PD-1, and CD8 expression in patients with thymomas and TC in the largest cohort study to date and analyzed the clinicopathological significance of this expression. We reviewed published studies on PubMed related to PD-L1 expression in TETs and summarized them in Table 5. Briefly, 18 studies were published in English, and the subjects examined in these studies ranged from 12 to 101 patients for thymoma and 3 to 69 patients for thymic carcinoma. The most frequent antibody evaluated was E1L3N (*n* = 6), followed by SP142 (*n* = 5), 22C3 (*n* = 1), and SP263 (*n* = 1). The interpretation criteria for PD-L1 in TETs have not yet been determined, and variable criteria were used according to the proportion of positive cells, intensity, or both. PD-L1 expression ranged from 18 to 92% in thymoma and 36.2 to 100% in TC. High PD-L1 expression was correlated with higher Masaoka-Koga staging in six studies (21, 22, 27, 32, 34, 36) and associated with WHO classification of type B and/or thymic carcinoma in eight publications (22, 23, 27, 31, 32, 34–36). Most studies did not report differences in OS between the high and low PD-L1 expression groups (*n* = 11) (16, 22, 24–27, 29, 30, 32, 33, 35), and three studies reported worse OS in the high PD-L1 expression group (21, 36, 37). In contrast, two studies reported better OS in the high PD-L1 expression group (24, 28).

**Table 5:** Summary of published series of PD-L1 expression in thymic epithelial tumors.

	**References**	**Number of patients**	**Antibody**	**Cut-off**	**PD-L1 positivity (%)**	**Significant parameter** **(ref. high PD-L1 expression)**	**Outcomes** **(ref. high PD-L1 expression)**
1	Padda et al. (21)	Thymoma: 65, TC: 4	Clone 15, Sino Biological	Score 3, intensity (0–3)	Thymoma: 44 (68%) TC: 3 (75%)	Younger age, high stage, incomplete resection, aggressive histology	Worse OS
2	Katsuya et al. (22)	Thymoma: 101, TC: 38	E1L3N, Cell signaling	≥3 (1%), H-score (0–300),	Thymoma: 22 (23%) TC: 26 (70%)	Thymic carcinoma	No significance
3	Yokoyama et al. (23)	Thymoma: 82, TC: 0	ERP1161 (Abcam)	≥38%, proportion	Thymoma: 44 (53.7%) TC: NA	Type B2 and B3 histology High stage (III and IV),	No significance
4	Yokoyama et al. (24)	Thymoma: 0, TC: 25	ERP1161 (Abcam)	≥20, H-score (0–300),	Thymoma: NA TC:20 (80%)	None	Better OS
5	Katsuya et al. (25)	Thymoma: 12, TC: 18	E1L3N, Cell signaling	≥1, H-score (0–300),	Thymoma: 6 (67%) TC: 7 (41%)	None	No significance
6	Marchevsky and Walts (26)	Thymoma: 38, TC: 8	SP142	≥1+, Semiquantified, (0–3+; 1, 5, and 20%)	Thymoma: 35 (92%) TC: 4 (50%)	NA	Not evaluated
7	Weissferdt et al. (16)	Thymoma: 74, TC: 26	E1L3N, Cell signaling	≥5%, TPS	Thymoma: 47 (64%) TC: 14 (54%)	None	No significance
8	Tiseo et al. (27)	Thymoma: 87, TC: 2	E1L3N, Cell signaling	≥3 (1%), H-score (0–300),	Thymoma: 15 (18%) TC: 13 (65%)	Thymic carcinoma High stage	No significance
9	Arbour et al. (28)	Thymoma: 12, TC: 11	E1L3N, Cell signaling	≥25%, TPS	Thymoma: 11 (91.7%) TC: 4 (36.3%)	None	Better OS
10	Owen et al. (29)	Thymoma: 32, TC: 3	22C3	≥3, Semiquantified, (0–5)	Thymoma: 26 (81.0%) TC: 3 (100%)	None	No significance
11	Duan et al. (30)	Thymoma: 13, TC: 20	CD274(ab58810), Abcam	≥6, Semiquantified^†^, (0–12)	Thymoma: 6 (46.2%) TC: 13 (65%)	None	No significance
12	Guleria et al. (31)	Thymoma: 84, TC: 0	SP263	≥25%, TPS	Thymoma: 69(82.1%), TC: NA	Type B histology (94.2%)	No significance in OS and DFS
13	Wei et al. (32)	Thymoma: 100, TC: 69	E1L3N, Cell signaling	Strong intensity or ≥50% in moderate intensity	Thymoma: 36 (36%), TC: 25 (36.2%)	High stage and type B in thymoma, No significance in TC	No significance in OS and DFS
14	Bedekovics et al. (33)	Thymoma: 29, TC: 7	SP142	≥50%, TPS, ≥10% IC	Thymoma: 9 (31.0%) in TPS, TC: 1 (14.3%) in TPS,	Low stage	No significance in OS and DFS
15	Chen et al. (34)	Thymoma: 50,TC: 20	SP142	≥3 (1%), H-score (0–300),	Thymoma: 24(48.0%), TC: 14(70%)	Type B3 and TC (76.6%), High stage, RTx and CTx	Not evaluated
16	Bagir et al. (35)	Thymoma: 38,TC: 6	CD274/PD-L1, (AM2653/AF-N)	≥5%, TPS	Thymoma: 31(81.5%), TC: 5 (83.3%)	Type B3 and TC	No significance in OS and DFS
17	Hakiri et al. (36)	Thymoma: 81,TC: 0	SP142	≥1%, TPS	Thymoma: 22(27.0%), TC: NA	Type B2 and B3 histology High stage (III and IV), high SUV	Worse in OS
18	Funaki et al. (37)	Thymoma: 0,TC: 43	SP142	≥50%, TPS	Thymoma: NA TC: 26 (60.5%)	Epithelial-mesenchymal transition	Worse in OS and DFS

† The percentage of positive tumor cells divided into five groups (5, 25, 50, and 70%; 0–4) and multiplied into intensity (1–3), ranging from 0 to 12.

The stage recorded according to Masaoka-Koga stage.

*TC, thymic carcinoma; TPS, tumor proportion score; ref, reference parameter; OS, overall survival; DFS, disease-free survival; RTx, radiation therapy; CTx, chemotherapy; SUV, standard uptake value; NA, not available; IC, positive immune cells; ref, references*.

Immunopositivity for PD-1 in thymoma ranged from 44 to 81% in thymomas and 23 to 47% in TC (16, 25, 26, 29), indicating a trend of much lower expression of PD-1 in TC than in thymomas. However, our study identified similar frequencies of PD-1 expression between thymomas (51.9%) and TC (55.0%). Moreover, Katsuya et al. (25) showed similar PD-1 expression in TC and thymomas (47% vs. 44%). No survival benefit in the PD-1 high expression group was observed in thymomas or TC in some studies (16, 25, 26, 29); our results were also similar. Weissferdt et al. (16) showed that PD-1 positive cases were associated with higher stage in TC; however, most studies revealed that PD-1 positivity was not associated with any parameters in thymomas or TC (25, 26, 29). We found that high PD-1 expression was correlated with lower Masaoka-Koga stage (*p* = 0.012) and absence of MG (*p* < 0.001) in thymoma, but, as in previous studies, no correlations with parameters were observed in TC.

Recently, the US Food and Drug Administration (FDA) approved the use of four anti-PD-1/PD-L1 drugs, including pembrolizumab, nivolumab, atezolizumab, and durvalumab, for the treatment of NSCLC, according to different corresponding immunohistochemical assays as companion or complementary diagnostic tests. In other words, each assay has a specific cutoff value for positive tumor cells, and the percentage may differ depending on whether the treatment was the first chosen treatment, even for the same drug in NSCLC. In addition, when an assay, such as SP263, is applied for two drug prescriptions, different cutoffs may be applied, depending on the drug. In Korea, the SP263 assay uses two different cut-offs for NSCLC: 25% for durvalumab (38) and 10% for nivolumab. These inconsistent cutoffs are not proven by clinical trials, except for durvalumab (38). However, a special situation that coincides with the national insurance policy in Korea causes pathologists to focus on insurance policies related with PD-L1 treatment. Several studies have shown that high PD-L1 expression is correlated with a better response to anti-PD-L1 therapy in NSCLC (7, 39, 40). In TETs, it may be expected that high PD-L1 expression would lead to a better response to anti-PD-L1 treatment, such as in NSCLC; however, the limited clinical trials of anti-PD-L1 therapy in TETs (10, 11) have shown controversial results. In a previous study, Giaccone et al. (10), using the 22C3 antibody for PD-L1, with a cutoff of more than 50%, revealed a better response in the high PD-L1 expression group, whereas Kim et al. (11) reported no significant response difference according to PD-L1 expression, with the same antibody and cutoff. Thus, the role of PD-L1 expression in TETs as a predictive marker for anti-PD-L1 treatment is still somewhat unknown.

As the cutoff of SP263 is not yet proven by clinical trials in TETs, we determined the cut-off as 50% because it is more highly expressed in TETs than in NSCLC. We found that the average TPS of 753 patients with NSCLC for SP263 was 25.02 ± 33.14 (data not shown), whereas it was 38.06 ± 32.35 in TETs. In addition, clinicopathologic correlations were more highly reflected at a cutoff of 50% than 25% (Supplemental Table 2, Supplemental Figure 2). The results obtained with the 25% cut-off were very similar to those of Guleria et al.; WHO classification of type B was correlated with PD-L1^high^ (31). Thus, an accurate pathologic diagnosis is crucial.

Several studies have reported the interchangeability of anti-PD-L1 antibodies in NSCLC; moreover, it has been reported that positive rates for PD-L1 were similar with high concordance under assay-specific cutoffs, except for SP142 (15, 41–43). Additionally, Sakane et al. (44) conducted a comparative study of PD-L1 immunohistochemical assays in thymic carcinoma, revealing results similar to those in NSCLC. Previous studies primarily used E1L3N clones in TETs (16, 22, 25, 27, 28, 32). Interestingly, SP263 IHC was deemed to be superior due to its strong staining intensity and higher sensitivity compared to E1L3N in NSCLC (45). We chose SP263 for the following reasons: first, we were unable to purchase the 22C3 antibody for research use because, at the time of our study, 22C3 was permitted only for use in companion diagnosis support by a global drug company in Korea; second, the positive rate for SP142 was very low in NSCLC; third, we considered it useful to choose a clinically validated and druggable antibody.

Our study demonstrated the highest PD-L1 immunopositivity in thymomas (95.5%, more than 1% cut-off) of all previous studies (16, 21–37). Thymomas are known to be rare epithelial tumors that occur more frequently in Asian patients, exhibiting higher staging and more frequent type B3 histology (46). Our study homogenously consisted of Korean patients and many cases of type B3 were included (36.5%).

A higher number of CD8^+^ TILs has been associated with better OS in melanoma, NSCLC, colorectal, breast, and ovarian cancers (1–3). Duan et al. (30) revealed that CD8^high^ TILs were associated with better OS in advanced thymic carcinoma; however, our study revealed no survival benefit in both OS (*p* = 0.351, log-rank test) and DFS (*p* = 0.913, log-rank test). In addition, none of the CD8/PD-L1 combination groups showed any survival benefit in OS (*p* = 0.509, log-rank) and DFS (*p* = 0.562, log-rank) in our study. Thus, the microenvironment of TETs may determine more than just CD8 activity; accordingly, more complex associations of immune cells could be considered.

Intra-tumoral and inter-tumoral heterogeneity of PD-L1 expression has been reported in NSCLC, with regard to the sampling issue (whole tumor section vs. biopsy or TMA) and location (primary or metastasis) (47). Gniadek et al. (47) compared 4 TMA cores from 150 formalin-fixed paraffin-embedded tissues of resected primary cancers and found, in many cases, substantial inconsistencies in the percentages of cells staining positive for PD-L1 among the different TMA cores. Our study also revealed inconsistencies, in many cases, among three TMA cores from the same sample, and we calculated the average TPS. Only one study has considered tumor heterogeneity in examining PD-L1 in TETs (29).

Our immunogenomic analysis by unsupervised clustering analysis revealed that the immune profiles of thymomas and TC were significantly different and that PD-L1 mRNA expression was positively correlated with PD-L1 protein expression (Spearman correlation analysis, *r* = 0.826, *p* < 0.001). In addition, the median PD-L1 mRNA level was higher in type B3 and TC than in the other thymomas, supporting the result that PD-L1^high^ expression by IHC correlated with type B3 in thymoma. Chen et al. (32) performed immunogenomic analysis of PD-L1 expression level in TETs using the TCGA database; their results were similar to ours—PD-L1 protein expression was correlated with PD-L1 mRNA expression level and PD-L1 mRNA expression level differed (*p* = 0.0419) between TC (median 9.39) and thymomas (median 5.68), suggesting that high PD-L1 expression was considerably correlated with TET malignancy.

To the best of our knowledge, this is the largest study published to date that focuses on a homogenous racial population. We believe that this study will provide useful information for the application of therapeutic drugs. Nevertheless, our study has several limitations: first, the cohort contained patients that underwent surgical resection of an adequate specimen for TMA, excluding inoperable cases. Second, this study was performed using TMA rather than whole tumor sections; thus, the possibility of false negative or positive results remains, despite the fact that we attempted to overcome this problem by making TMAs in triplicate.

In conclusion, our study revealed that PD-L1 expression was frequent in TETs and associated with high Masaoka-Koga staging; moreover, WHO classification of type B3 was correlated with poor prognosis. In addition, PD-L1 expression is an independent prognostic factor in thymoma. Our results suggest the importance of appropriate patient selection in anti-PD-L1 therapy in TETs and the accurate pathologic diagnosis of TETs is essential to predict the patient's prognosis.

## Data Availability Statement

This manuscript contains previously unpublished data. The name of the repository and accession number(s) are not available.

## Ethics Statement

This study was approved by the Ethics Committee of the Asan Medical Center (approval number: 2015- 965). The patients/participants provided their written informed consent to participate in this study.

## Author Contributions

JS and SJ: conception and design. JS, JK, and DK: development of the methodology. C-MC and HK: data acquisition. JS and DK: data analysis. JS, DK, and SJ: writing, review, and/or revision of manuscript. All contributors met the criteria for authorship and approved the final manuscript.

### Conflict of Interest

The authors declare that the research was conducted in the absence of any commercial or financial relationships that could be construed as a potential conflict of interest.
