# Supplementary material for: Clinicopathologic Significance and Immunogenomic Analysis of Programmed Death-Ligand 1 (PD-L1) and Programmed Death 1 (PD-1) Expression in Thymic Epithelial Tumors
Source: Front Oncol. 2019 Oct 15;9:1055. doi: 10.3389/fonc.2019.01055 (PMC6803548; doi:10.3389/fonc.2019.01055)
Supplement: Supplementary file 1 [file Data_Sheet_1.docx]

Supplementary Material

**Supplement Figure 1. Survival analysis according to combination of PD-L1 and PD-1 expression in thymomas**. PD-L1 ^low^ / PD-1 ^high^ expression group shows good prognosis in OS (b, p=0.009) compared to PD-L1 ^high^ / PD-1 ^low^ , but no significance is observed in DFS (a, p=0.082).

**Supplement Figure 2.** Survival analysis by Kaplan-Meier in thymomas. When the cutoff is 25%, PD-L1 ^high^  group shows the shorter disease free survival compared to PD-L1 ^low^ group (a, p=0.011), but no survival differences between PD-L1 ^high^ and PD-L1 ^low^ groups in OS (b, p=0.069) is observed.

**Supplement Figure 3.** Representative images of thymic epithelial tumor and PD-L1 (Sp263) IHC. (A) thymoma type A (H &E, original magnification x200 ) (B) PD-L1 of type A (immunohistochemistry, original magnification x200). (C) Thymic carcinoma (H &E, original magnification x200 ). (D) PD-L1 of thymic carcinoma (immunohistochemistry, original magnification x200).

**Supplement Table 1**. Patient demographics for transcriptome sequencing

| **Parameters** | **N (n=42, %)** |
| --- | --- |
| **Age (median, year)** | 54 (range, 26-80) |
| **Gender** |  |
| Male | 26 (61.9%) |
| Female | 16 (38.1%) |
| **Masaoka-Koga stage** |  |
| I | 11 (26.2%) |
| IIa | 14 (33.3%) |
| IIb | 2 (4.8%) |
| III | 12 (28.6%) |
| IVa | 2 (4.8%) |
| IVb | 1 (2.4 %) |
| **WHO classification** |  |
| A (Atypical) | 1 (2.4%) |
| AB | 4 (9.5%) |
| B1 | 2 (4.8 %) |
| B2 | 1 (2.4%) |
| B3 | 16 (38.1%) |
| Thymic carcinoma | 16 (38.1%) |
| Micronodular type | 2 (4.8%) |
| **Myasthenia gravis** |  |
| Yes | 10 (23.8%) |
| No | 32 (6.2%) |
| **Neoadjuvant Tx** |  |
| Yes | 5 (11.9%) |
| No | 37 (88.1%) |
| **Adjuvant Tx** |  |
| No | 13 (31.0%) |
| CTx | 4 (9.5%) |
| RTx | 25 (59.5%) |

Abbreviations: Tx, treatment; CTx, chemotherapy; RTx, radiation therapy.

**Supplement Table 2**. Correlation between PD-L1 and clinicopathologic parameters (cutoff , 25%)

| Parameters | Thymoma (N=302) | |  | Thymic carcinoma (N=60) | |  |
| --- | --- | --- | --- | --- | --- | --- |
|  | High (n=175, %) | Low (n=127, %) | P value | High (n= %) | Low  (n=, %) | P value |
| **Age**† | 52.3 ± 13.42 | 52.4 ± 12.54 | 0.956 | 56.73 ±  9.83 | 55. 09±  12.16 | 0.576 |
| **Gender** |  |  | 0.115 |  |  | 0.548 |
| Male | 96 (54.9%) | 58 (45.7%) |  | 18(69.2%) | 21(61.8%) |  |
| Female | 79 (45.0%) | 69 (54.3%) |  | 8(30.8%) | 13(38.2%) |  |
| **Masaoka stage** |  |  | 0.001 |  |  | 0.963 |
| I | 93(53.1%) | 96(75.6%) |  | 7(26.9%) | 8(23.5%) |  |
| IIa | 32 (18.3%) | 19(15.0%) |  | 4(15.4%) | 5(14.7%) |  |
| IIb | 13 (7.4%) | 5 (3.9%) |  | 1(3.8%) | 295.9%) |  |
| III | 30 (17.1%) | 7 (5.5%) |  | 8(30.8%) | 12(35.3%) |  |
| IVa | 1 (0.6%) | 0 (0%) |  | 2(7.7%) | 1(2.9%) |  |
| IVb | 6 (3.4%) | 0 (0%) |  | 4(15.4%) | 6(17.6%) |  |
| **WHO classification** |  |  | <0.001 |  |  |  |
| A | 11 (6.3 %) | 21 (16.5%) |  |  |  |  |
| AB | 24(13.79%) | 66 (52.0%) |  |  |  |  |
| B1 | 23(13.1 %) | 20 (15.7%) |  |  |  |  |
| B2 | 51 (29.1 %) | 14 (11.0%) |  |  |  |  |
| B3 | 66 (37.7%) | 6 (4.7%) |  |  |  |  |
| **Size** |  |  | 0.008 |  |  | 0.228 |
| <5 cm | 71 (40.6%) | 33 (26.0%) |  | 4(15.4%) | 11(32.4%) |  |
| ≥ 5 cm | 104 (59.4%) | 94(74.0%) |  | 22(84.6%) | 23(67.6%) |  |
| **Capsule formation** |  |  | 0.013 |  |  | 0.688 |
| present | 74 (42.3%) | 72 (56.7%) |  | 2(7.7%) | 5(14.7%) |  |
| absent | 101(57.7%) | 55 (43.3%) |  | 24(92.3%) | 29(85.3%) |  |
| **Capsule invasion** |  |  | <0.001 |  |  | 0.773 |
| present | 63 (36.0%) | 21 (16.5%) |  | 17(65.4%) | 21(61.8%) |  |
| absent | 112 (64.0%) | 106(83.5%) |  | 9(34.6%) | 13(38.2%) |  |
| **LN metastasis** |  |  | 0.227 |  |  | 0.781 |
| present | 2 (1.1%) | 0 (0%) |  | 2(7.7%) | 2(5.9%) |  |
| absent | 173 (98.9%) | 127 (100%) |  | 24(92.3%) | 32(94.1%) |  |
| **Distant metastasis** |  |  | 0.221 |  |  | 0.643 |
| present | 7(4.0%) | 2 (1.6%) |  | 6(23.1%) | 6(18.2%) |  |
| absent | 168 (96.0%) | 125 (98.4%) |  | 20(76.9%) | 27(81.8%) |  |
| **Myasthenia gravis** |  |  | <0.001 |  |  | None |
| present | 61 (34.9%) | 12 (9.4%) |  | 0(0%) | 0(0%) |  |
| absent | 114 (65.1%) | 115 (90.6%) |  | 26(100%) | 34(100%) |  |
| **Neoadjuvant Tx** |  |  | 0.344 |  |  | 0.611 |
| No | 161 (92.0%) | 122 (96.1%) |  | 16(61.5%) | 25(73.5%) |  |
| CTx | 12(6.9 %) | 4(3.1%) |  | 9(34.6%) | 8(23.5%) |  |
| RTx | 2(1.1% ) | 1(0.8%) |  | 1(3.8%) | 1(2.9%) |  |
| **Adjuvant Tx** |  |  | <0.001 |  |  | 0.355 |
| No | 100 (57.1%) | 102 (80.3%) |  | 6(23.1%) | 11(32.4%) |  |
| CTx | 3 (1.7%) | 1 (0.8%) |  | 3(11.5%) | 7(20.6%) |  |
| RTx | 72 (41.1%) | 24 (18.9%) |  | 17(65.4%) | 16(47.1%) |  |

† Analyzed with T-test.

‡ Neoadjuvant treatment includes chemotherapy or radiation therapy.

Abbreviations: Tx, treatment; CTx, chemotherapy; RTx, radiation therapy.
