# Supplementary material for: Clinicopathologic Significance and Immunogenomic Analysis of Programmed Death-Ligand 1 (PD-L1) and Programmed Death 1 (PD-1) Expression in Thymic Epithelial Tumors
Source: Front Oncol. 2019 Oct 15;9:1055. doi: 10.3389/fonc.2019.01055 (PMC6803548; doi:10.3389/fonc.2019.01055)
Supplement: Supplementary file 2 [file Presentation_1.PPTX]

## Slide 1
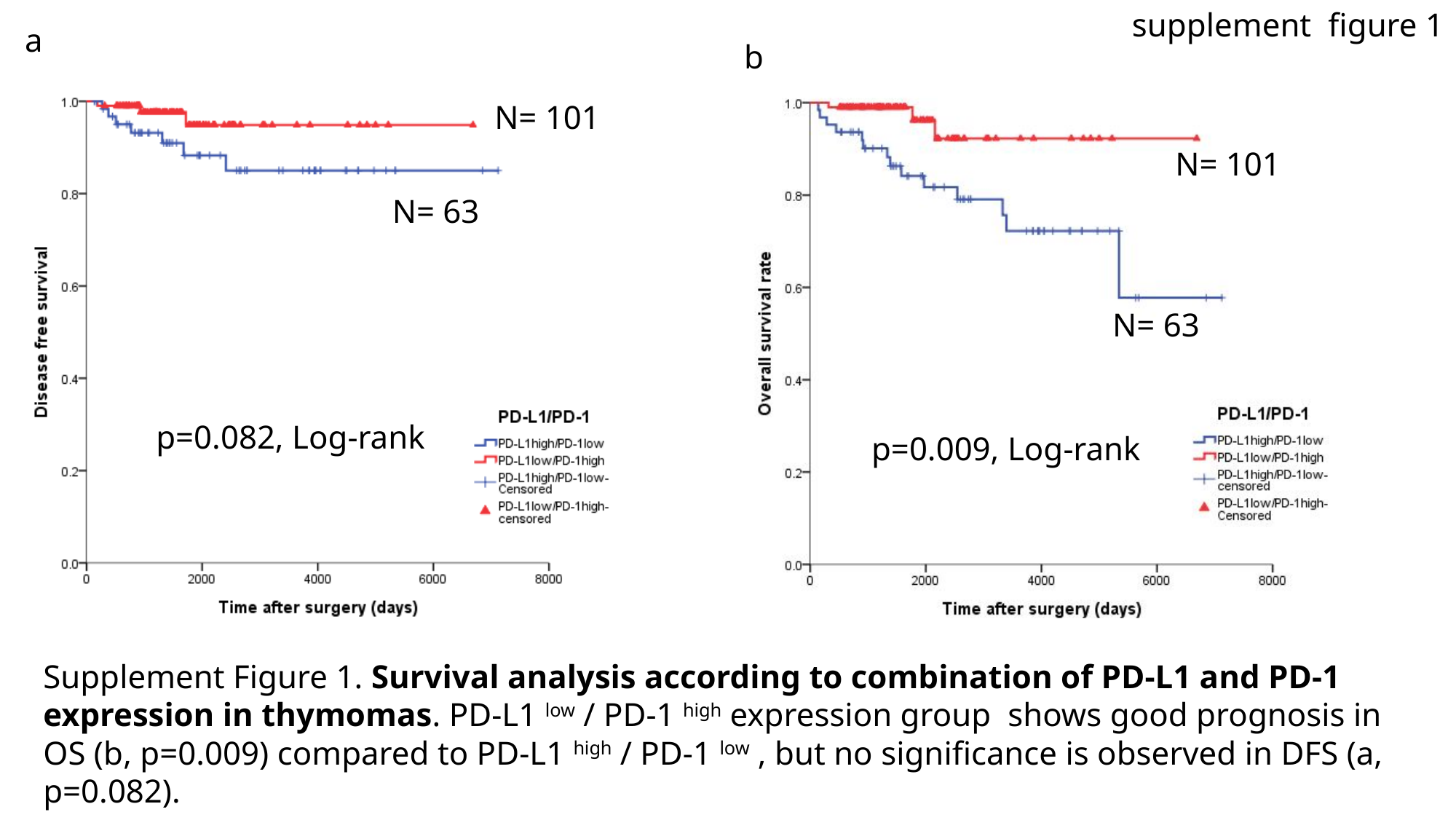

supplement figure 1
a
b
N= 101
N= 101
N= 63
N= 63
p=0.082, Log-rank
p=0.009, Log-rank
Supplement Figure 1. Survival analysis according to combination of PD-L1 and PD-1 expression in thymomas. PD-L1 low / PD-1 high expression group shows good prognosis in OS (b, p=0.009) compared to PD-L1 high / PD-1 low , but no significance is observed in DFS (a, p=0.082).

## Slide 2
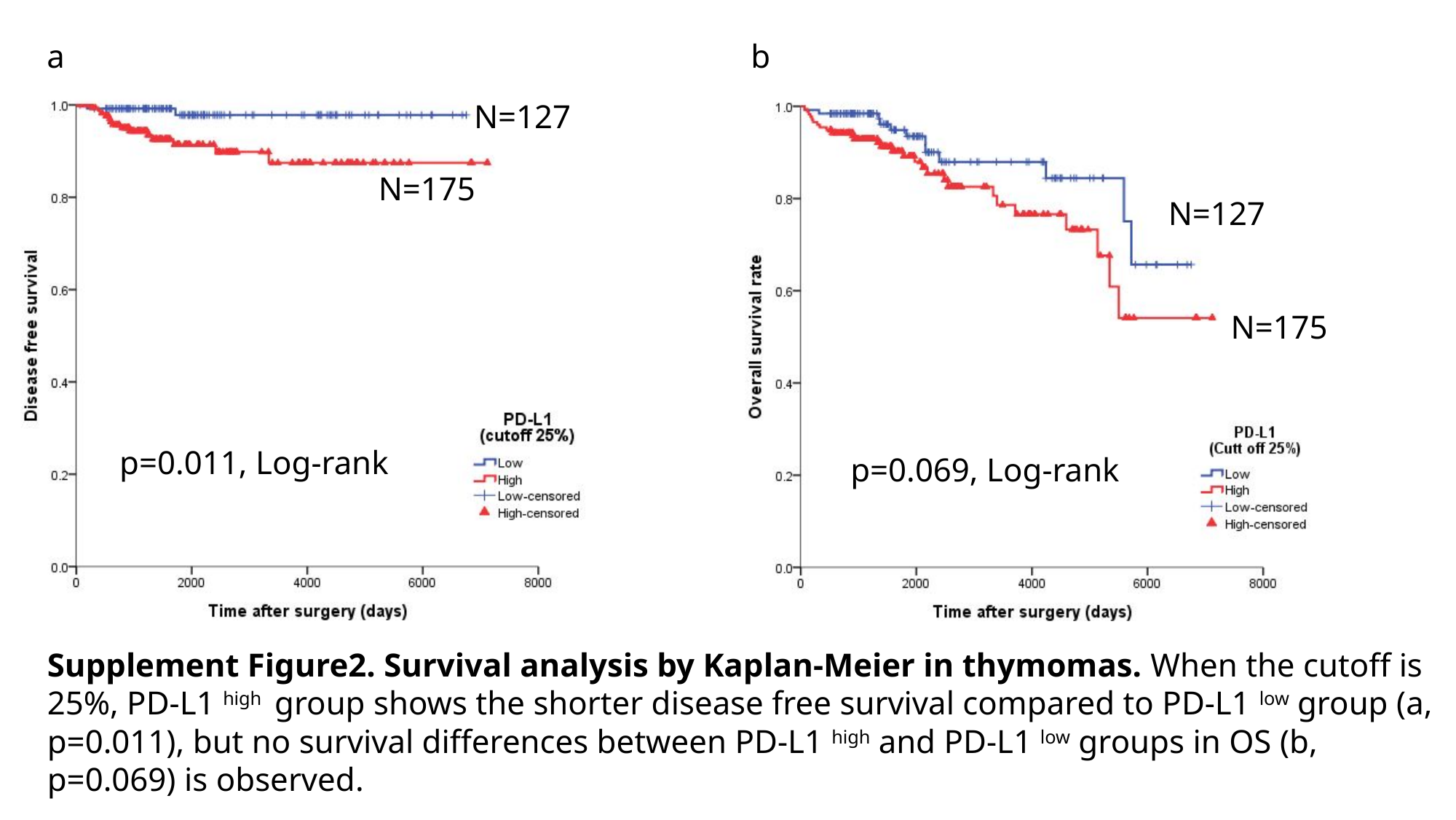

a
b
N=127
N=175
N=127
N=175
p=0.011, Log-rank
p=0.069, Log-rank
Supplement Figure2. Survival analysis by Kaplan-Meier in thymomas. When the cutoff is 25%, PD-L1 high group shows the shorter disease free survival compared to PD-L1 low group (a, p=0.011), but no survival differences between PD-L1 high and PD-L1 low groups in OS (b, p=0.069) is observed.

## Slide 3
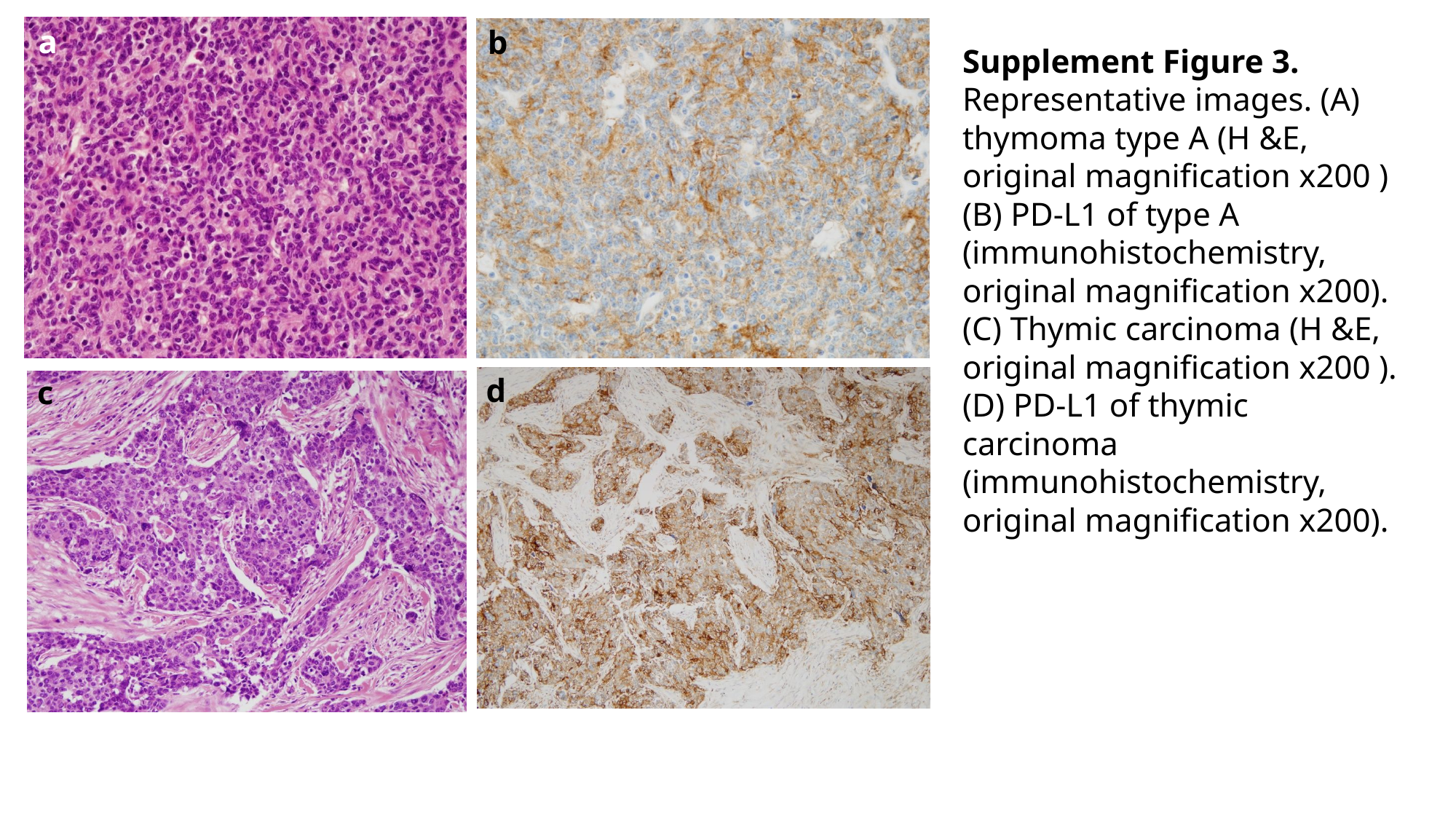

a
b
Supplement Figure 3. Representative images. (A) thymoma type A (H &E, original magnification x200 ) (B) PD-L1 of type A (immunohistochemistry, original magnification x200). (C) Thymic carcinoma (H &E, original magnification x200 ). (D) PD-L1 of thymic carcinoma (immunohistochemistry, original magnification x200).
d
c
